# Evaluation of Different Sensor Systems for Classifying the Behavior of Dairy Cows on Pasture

**DOI:** 10.3390/s24237739

**Published:** 2024-12-03

**Authors:** Barbara Pichlbauer, Jose Maria Chapa Gonzalez, Martin Bobal, Christian Guse, Michael Iwersen, Marc Drillich

**Affiliations:** 1 Center for Veterinary Systems Transformation and Sustainability, Clinical Department for Farm Animals and Food System Science, University of Veterinary Medicine, 1210 Vienna, Austria; barbara.pichlbauer@vetmeduni.ac.at (B.P.); josemaria.chapagonzalez@hochschule-rhein-waal.de (J.M.C.G.); resuscitationpicture@gmail.com (M.B.); christian.guse@vetmeduni.ac.at (C.G.); marc.drillich@fu-berlin.de (M.D.); 2 Unit for Reproduction Medicine and Udder Health, Faculty of Veterinary Medicine, Freie Universität Berlin, 14163 Berlin, Germany

**Keywords:** evaluation, sensor technology, monitoring, behavior, cattle, grazing

## Abstract

Monitoring animal behavior using sensor technologies requires prior testing under varying conditions because behaviors can differ significantly, such as between grazing and confined cows. This study aimed to validate several sensor systems for classifying rumination and lying behaviors in cows on pasture under different environmental conditions, compare the sensors’ performance at different time resolutions, and evaluate a correction algorithm for rumination data. Ten Simmental dairy cows were monitored on pasture, each simultaneously equipped with an ear-tag accelerometer (ET), two different leg-mounted accelerometers (LMs), and a noseband sensor (NB). Indirect visual observations using drone-recorded video footage served as the gold standard for validation. The concordance correlation coefficient (CCC) for rumination time was very high for both the ET and NB (0.91–0.96) at a 10 min time resolution. Applying the correction algorithm to 1 min data improved the CCC for the NB from 0.68 to 0.89. For lying time, the CCC was moderate for the ET (0.55) but nearly perfect for both LMs (0.99). In conclusion, both sensors evaluated for classifying rumination are suitable for cows on pasture. We recommend using a correction algorithm for 1 min NB data. For the measurement of lying time, the LMs significantly outperformed the ET.

## 1. Introduction

During the past few decades, modern dairy farming has undergone significant changes, driven in part by rapid technological advancements. These changes have led to the emergence of a field known as precision livestock farming (PLF). In general, the adoption of digital automation technology in agriculture has been driven by two main factors: rising food demand and decreasing natural resources [1]. Concurrently, especially in Europe, public awareness has increased regarding issues such as food quality and safety, traceability, and animal welfare. Dairy farms that incorporate grazing into their management practices are not only perceived as more natural by consumers but can also offer benefits in terms of animal health, such as improved hoof health [2], enhanced animal welfare, increased product quality, and greater global sustainability, provided they are implemented correctly [3]. As a result, grazing-based dairy farming may gain greater significance in the future.

PLF technologies present a promising approach to addressing these challenges by offering new opportunities to automatically classify animal behavior and monitor changes at both the individual and herd levels. Rumination times and lying behavior are critical parameters that can help monitor physiological and pathological changes in cows. For example, decreased rumination and lying times can be useful predictors of estrus [4], while reduced lying times can be observed in cows with mastitis. Conversely, lame cows tend to spend more time lying down than healthy controls [5]. Automated monitoring of rumination time and activity during the early postpartum period can also be valuable in detecting metabolic and digestive diseases in dairy cows [6].

There are several approaches to categorize automated monitoring systems in PLF, for example, according to their position in relation to the animal (on-cow, in-cow, and off-cow sensors), their invasiveness (invasive, minimally invasive, and non-invasive), or the technology employed (e.g., accelerometers, temperature sensors, pressure sensors, and camera sensors). In cows, wearable sensors can be mounted in various ways, such as on a collar, halter, or ear-tag; inside the reticulorumen; or intravaginal. Using accelerometers for behavioral monitoring is a widespread approach [7], with specific algorithms classifying behavior based on acceleration patterns. However, these sensors need to be attached to specific positions on the animal, which can be rated as invasive to a certain extent and are therefore currently under discussion. Off-cow sensors include systems to measure environmental parameters. Moreover, recent developments, such as computer vision technologies, aim to classify the behavior of several animals simultaneously, without interfering with them at all.

Given the availability of various sensor systems capable of classifying lying and rumination behavior, advancements in research on these behaviors can be readily applied on many farms utilizing automated monitoring systems. However, before these systems are widely adopted, it is crucial to independently validate the technical devices and sensor systems to ensure their reliability and accuracy. Extensive research has already been conducted to validate various sensor systems under different conditions, as reviewed by Chapa et al. [7] and Stygar et al. [8].

The SMARTBOW system (Smartbow GmbH/Zoetis LLC, Weibern, Austria) is an ear-tag accelerometer (ET) and has demonstrated strong performance in classifying rumination and as a tool for estrus detection in barn environments [9,10,11]. Additionally, this ET has been validated for detecting grazing behavior in dairy cows [12]. However, to our knowledge, no studies have evaluated its effectiveness in monitoring rumination and lying times in dairy cows on pasture.

Several studies have evaluated HOBO-loggers (HOBO Pendant G logger; Onset Computer Corporation, Bourne, MA, USA) as leg-mounted accelerometers (LMs) for classifying the standing and lying positions of cows and calves under indoor conditions [13,14,15]. While this leg-mounted accelerometer (LM1) has been used in research involving grazing cows [16], it has not been specifically validated for use on pasture. When working with sensor systems, it is beneficial to evaluate them under different conditions.

The RumiWatch system (ITIN + HOCH GmbH, Liestal, Switzerland) is a widely used research tool in PLF and consists of two independent components: a halter with an integrated noseband sensor (NB) and a pedometer (LM2). This system is capable of classifying various behavioral categories and has been scientifically validated for use under both confined [17] and pasture conditions [18]. Because of its extensive testing by numerous research groups, classification algorithms have been adapted, resulting in different versions of the converter software (currently available: V.0.7.3.2, V.0.7.3.36, V.0.7.4.5, and V.0.7.4.13). Despite these advancements, recent studies have continued to use older software versions. For example, Pereira et al. [19] evaluated V.0.7.3.36 and recommended the NB and LM2 as a reference for other systems in detecting grazing, rumination, and lying behavior. Other researchers have compared different converter versions of the NB, evaluating their performance under confined versus grazing conditions (V.0.7.3.36 and V.0.7.4.13 [20]), under thermoneutral versus heat stress conditions (V.0.7.3.2 and V.0.7.3.36 [21]), and at different time resolutions of raw data classification (V.0.7.4.5 [22]). Li et al. [23] assessed the latest version (V.0.7.4.13) against visual observations and found a high correlation for the detection of rumination chews. Unlike previous versions, V.0.7.4.13 no longer offers behavior classification at a 1 min time resolution. Despite the widespread use of the NB and LM2, a direct comparison of the three most recent software versions has not been conducted. Therefore, we investigated the performance of these versions of the NB and LM2 (V.0.7.3.36, V.0.7.4.5, and V.0.7.4.13) in classifying the rumination and lying times of cows at different time resolutions using the same dataset. These versions are hereafter referred to as RWC1, RWC2, and RWC3, respectively.

Acknowledging the increasing importance of climate change, we also considered the possibility of the varying performance of technical tools under changing environmental conditions in grazing dairy cows. A recent review [24] reported that environmental conditions, such as heat stress, can change animal behavior in terms of reduced lying, feeding, and rumination time; increased water intake; and shade-seeking and insect avoidance behavior, and may even provoke aggressive behaviors around waterers, for example.

The objectives of this study were threefold: first, to evaluate the performance of an ET for detecting rumination and lying behavior and to validate two LMs for classifying lying behavior on pasture using indirect visual observation (IVO) as the gold standard across different time resolutions; second, to directly compare the three most recent converter versions of the RumiWatch system; and, third, to gain insights about the impact of differing ambient temperatures on the automated classification of behaviors in grazing cows. To the best of our knowledge, this is the first study to evaluate the performance of several sensor technologies for use in cows on pasture under changing environmental conditions. The key conclusions are that both the ET and the NB are suitable for measuring rumination time on pasture, and that both LMs are highly effective for detecting lying behavior in grazing cows.

## 2. Materials and Methods

### 2.1. Study Farm

The study was conducted from June to October 2020 at the Teaching and Research Farm (VetFarm) of the University of Veterinary Medicine Vienna, Austria. This study was part of a larger project on the use of various sensor technologies in grazing dairy cows, which spanned from 2020 to 2021. The farm housed approximately 80 Simmental dairy cows. The lactating cows were kept in a free-stall barn with cubicles and straw bedding. They were milked twice daily in a tandem milking parlor. In 2020, the herd’s energy-corrected milk yield, based on 4.0% butterfat and 3.4% protein, was 9308 kg. Dry cows were housed separately in another barn with deep-bedded straw. A total mixed ration was provided in the barn. Additionally, lactating cows grazed on pasture for several hours daily from May to October, with approximately 1.5 hectares of adjacent pasture available for their use.

### 2.2. Sensor Systems

#### 2.2.1. SMARTBOW System

This ET system comprises three main components: an accelerometer integrated into an ear-tag, receivers, and a farm server. For research purposes, acceleration data are collected at a frequency of 10 Hz, in contrast to the commercial system, which logs data at 1 Hz. The ear-tag wirelessly transmits the raw data to the receivers, which then relay the data to an on-farm server. Proprietary algorithms classify the raw data in real time into various behavioral categories, such as ruminating, standing, and lying [9,10,25]. For use on pasture, specially designed receiver stations with solar-powered batteries were available in this study. At the VetFarm, nine receiver stations were installed around a total pasture area of approximately 2.4 hectares. The average distance between the receivers along the pasture was 70 m, with a range of 42 to 101 m. These receivers were wirelessly connected to enable real-time communication with each other and to transmit raw data packages to the on-farm server. Further details about this ET system can be found in the report by Schweinzer et al. [11]. Pictures of the system and its implementation on the study farm are shown in Figure S1 of the Supplementary Materials.

#### 2.2.2. HOBO-Loggers

HOBO-loggers are data loggers equipped with built-in triaxial accelerometers and a gyroscope function to measure the tilt of the logger. The raw data, including acceleration and tilt data in the x-, y-, and z-axes, are stored in internal memory and can be retrieved using a proprietary optic USB interface (connector, Base U-44, and coupler). The logging interval can be customized prior to the start of a measurement series depending on the experiment’s specific requirements (maximum logging frequency: 100 Hz; maximum logging interval: 18 h, 12 min, 15 s). In this study, the frequency was set to 1 Hz in accordance with the standard operating procedure derived from Ito et al. [13]. The loggers were managed, and data were downloaded using HOBOware software (HOBOware Pro 3.7.21; Onset Computer Corporation, Bourne, MA, USA). The data loggers were wrapped in foam and secured with bandage material to the left hind legs of the study animals to capture their standing and lying times (see Figure S2 of the Supplementary Materials).

#### 2.2.3. RumiWatch System

This sensor system consists of two primary components: a NB integrated into a halter, and a pedometer, referred to as LM2 in this manuscript. The NB is an oil-filled tube with a pressure sensor embedded in the noseband, along with a triaxial accelerometer housed in a plastic box attached to one side of the cheek. The halter must be properly fitted to the cow’s head to ensure adequate pressure differences when the cow chews, which is essential for accurately detecting rumination time, eating time, drinking, and other behaviors. The NB is shown in Figure S3 of the Supplementary Materials.

The LM2, which contains an accelerometer secured inside a plastic box, can be attached with a strap to any of the cow’s legs (lateral metacarpus or metatarsus). It measures activity-related behaviors such as standing, lying, and walking. In this study, the pedometers were mounted on the cows’ right lateral metatarsus (see Figure S3 of the Supplementary Materials).

Although the halter and pedometer components of the system can be used independently, both are managed using the same software, RumiWatch Manager 2 (Version 2.2.0.0; ITIN + HOCH GmbH, Liestal, Switzerland). The system records raw data at a 10 Hz frequency. These data are stored on an internal micro-SD card and can be downloaded at the end of a measurement series via a cable connection. Two 3.6 V lithium-metal (Li-SOCL_2_) batteries power the halter and pedometer. A more detailed description of this sensor system is available in the report by Zehner et al. [17]. In this study, we focused on the behavioral classifications for rumination and lying time.

### 2.3. Study Design

Ten lactating cows were selected on the basis of their reproductive status (inseminated or already confirmed pregnancy) to minimize disruptions from farm management procedures, such as breeding and pregnancy checks, during the study. At the start of the study, the enrolled cows were at 202 ± 41 days in milk and had undergone 2.2 ± 1.2 lactations (mean ± standard deviation). Each cow was simultaneously equipped with the three sensor systems to collect behavioral data, and their behavior was also recorded using a camera-equipped drone (DJI Phantom 4 Pro V 2.0; SZ DJI Technology Co., Shenzhen, China). IVO served as the gold standard for behavioral classification.

The required observation hours for this study were calculated using G*Power software (G*Power Version 3.1.9.2; Franz Faul, Kiel University, Kiel, Germany). To detect a small to medium effect size of 0.2 with a Type I error probability (α) of 0.05 and a power (1 − β) of 0.80, 191 h of animal observation were necessary.

Before the study began, the cows were gradually habituated to the sensor systems, the grazing regimen, and the drone flights over several non-consecutive weeks starting in May 2020. During the experimental periods, the group of enrolled cows was brought to pasture during four non-consecutive weeks throughout the grazing season. Each experimental period consisted of 4 days, from Monday to Thursday, during which the study group was moved to pasture in the morning between 8:00 AM and 9:30 AM local time (Central European Summer Time). Depending on the weather conditions (e.g., too hot or too windy for drone flights), the cows were brought back to the barn after 2.0 to 5.5 h. During the second week of the experiment, the cows were also moved to pasture after the evening milking, between 6:20 PM and 7:00 PM Central European Summer Time, for 2.0 to 2.5 h. For the rest of the day, the cows were housed indoors with the rest of the lactating herd.

The cows were fenced into smaller areas of approximately 0.3 hectares to facilitate simultaneous observation of all animals by the drone. A new strip of pasture was allocated to the animals depending on the availability of grass. Throughout the study, ambient temperature and relative humidity in the barn were measured using data loggers with built-in sensors (TinyTag Plus 2 TGP-4500; Gemini Data Loggers, Chichester, UK). For pasture, local weather data from the nearby official weather station in Berndorf (station 7641, officially calibrated according to the World Meteorological Organization) were available and used for analyses regarding the environmental conditions.

No further manipulation or habituation was necessary for using the ET in this study because the system was already in regular use at the VetFarm for general herd management purposes, such as estrus detection. The NB and LMs were mounted on the cows on the Sunday before the start of each experimental period and removed after the 4 consecutive measurement days. During these periods, the cows were examined twice daily after milking for potential bruises on their heads or legs caused by the sensors. The positions of the sensors were checked and adjusted if necessary.

All sensor systems were time-synchronized using Coordinated Universal Time as a reference. The simultaneous use of the different sensors on each cow was intended to ensure consistent conditions (both behavioral and environmental) across all systems, thereby reducing potential confounding factors.

#### Visual Observations

The drone equipped with an RGB camera provided sufficient flexibility to follow the small group of study cows on their respective allocated pastures. The size of the pasture was determined by the area that the camera could capture in a single frame, ensuring that a human observer could consistently identify the cows and their behaviors. Video footage was recorded in 4 K resolution using the H.265 video codec and stored as mp4 files during the flight on 128 GB UHS-I micro-SD cards (SanDisk Extreme R160/W90 micro SDXC; SanDisk, Western Digital Technologies, Inc., Milpitas, CA, USA). The drone pilots were trained in advance to capture all cows as closely as possible without disturbing their natural behavior. The drone’s theoretical maximum continuous flight time was 30 min according to the manufacturer, limited by the battery, with an additional 5 to 10 min required between flights to change the battery. Time synchronicity was maintained using Coordinated Universal Time as a reference, with the timestamp added to the subtitles of the video footage to facilitate later analysis of the recordings. For behavioral classification, two observers were trained in advance to use the ethogram shown in Table 1.

For the labeling process, the software Mangold^®^ Interact (Version 17.1.11, Mangold International GmbH, Arnsdorf, Germany) was used. In total, 94 video sequences were available. The mean analyzable portion of the video sequences was 17.85 ± 2.60 min. After excluding sequences that were not suitable for analysis because of video quality issues or data losses (mainly from the SBS), 69 sequences were deemed eligible for analysis. Of these, 12 were randomly selected to assess inter-rater reliability. The remaining 57 videos were divided, with each observer receiving a separate randomly allocated set of 27 videos. At the end of this selection and allocation process, 66 video sequences, with a total duration of 19 h, 54 min, and 3 s, were analyzable. To avoid biases, the observers were blinded throughout the entire selection and allocation process, which was conducted using R Statistical Software Version 4.0.4 [27] and the “caret 6.0-92” package [28]. Because the drone observation strategy allowed for the simultaneous capture of all 10 cows, the available video footage amounted to approximately 199 h in total.

### 2.4. Data Preparation

Sensor data from various sources, as well as data from IVOs, were uploaded to an Influx database (InfluxDB OSS v2.0). For data pre-processing, Python software, along with the pandas and NumPy packages (Python 3.7.9, pandas 1.3.5, and NumPy 1.21.5 [29]), was used. The pre-processed data were then resampled and merged with the InfluxDB.

The IVO data were recorded with a 1 s time resolution, starting from the moment the cows and their behaviors could first be identified in each video sequence. These data were resampled to 1 min and 10 min intervals to align with the sensor systems’ data at different time resolutions. A longer time interval was deemed unreasonable because of the limited length of the video sequences.

ET data were provided by the company as continuous data with 1 min time resolutions. Each minute was classified according to the predominant behavior as either “lying/standing” or “rumination/nothing”. These data were resampled to a 10 min resolution to enable a direct comparison across all sensor systems and IVO within a single dataset.

For the LM1, raw data were available at a 1 min time resolution and were classified into “standing” and “lying” using a threshold of 60° of vertical tilt, as described by Ito et al. [13]. Corrections of erroneous values, such as standing or lying bouts lasting <3 min, were made using a Python script (hereafter referred to as the correction algorithm (CA)) derived from the Excel macros published by Ledgerwood et al. [14]. This CA removes single events lasting ≤2 min, thereby eliminating erroneous classifications such as brief kicking movements that would otherwise be misclassified as 1 min of lying. The classified data were then resampled to a 10 min time resolution for direct comparison with the other systems.

Data from the NB and LM2 were available as raw data at a 10 Hz time resolution, stored in a proprietary file format (.rwu). These files were converted into classified .csv files using the three latest versions of the RumiWatch Converter (RWC) (ITIN + HOCH GmbH, Liestal, Switzerland): V0.7.3.36 (RWC1), V0.7.4.5 (RWC2), and V0.7.4.13 (RWC3). With RWC1 and RWC2, the data were classified into 1 min and 10 min time resolutions. However, RWC3 does not support a 1 min time resolution classification, so only 10 min time resolution data were available. Because there was no validity check for conversion at the 1 min time resolution [17], the same CA used for the LM1 was applied to the converted rumination data from RWC1 and RWC2 in a subsequent step. To enhance comparability with the ET, this CA was also applied to the 1 min time resolution data of the ET system.

### 2.5. Statistical Analyses

Statistical analyses were conducted using R Statistical Software Version 4.0.4 [27]. The packages “caret 6.0-92” [28], “chillR 0.72.4” [30], “DescTools 0.99.41” [31], “irr 0.84.1” [32], “misty 0.4.11” [33], and “tidyr 1.1.3” [34] were utilized for various analyses, while “Cairo 1.5-15” [35], “ggplot2” [36], and “showtext 0.9-5” [37] were used for graphical representation of the data. To assess the inter-rater reliability of the IVOs, Cohen’s kappa coefficient (κ) was calculated between the two independent observers. Histograms and the Shapiro–Wilk test were used to assess the normal distribution of sensor data across different time resolutions.

Lin’s concordance correlation coefficient (CCC) and Spearman’s rank correlation coefficient (r_S_) were calculated to evaluate the agreement between IVOs and each sensor system or converter version. These analyses were performed separately for lying and rumination behavior.

For categorical data at the 1 min time resolution, a confusion matrix was computed to determine the sensitivity (SE), specificity (SP), positive predictive value (PPV), and balanced accuracy (AC) for each system or converter version compared with IVO. SE was defined as the true positives (TPs) divided by the sum of TPs and false negatives (FNs). SP was defined as the true negatives (TNs) divided by the sum of false positives (FPs) and TNs. The AC was defined as the sum of sensitivity and specificity, afterwards divided by two. The formula for the PPV was as follows

PPV = (SE × prevalence)/((SE × prevalence) + ((1-SP) × (1-prevalence))),(1)

with prevalence defined as the sum of TPs and FNs divided by the sum of TPs, FPs, FNs, and TNs. For rumination behavior, these analyses were conducted both before and after applying the CA.

For numerical data at the 10 min time resolution, the coefficient of determination (R^2^) and root mean square error (RMSE) were calculated in addition to CCC and r_S_. For graphical analysis and comparison, lying and rumination times (min/10 min) from the sensor systems were plotted against IVO data, with the regression lines displayed. For trying to determine if weather-related factors influenced system performance (by affecting the animals’ behavior, for example), the temperature–humidity index (THI) was calculated using data from the local weather station in Berndorf and the formula described by Kendall and Webster [38]. The THI data were merged by timestamps with the sensor and IVO data (at a 10 min time resolution). Because cows can show signs of heat stress at a THI of ≥68 [39], this value was used as the threshold to subdivide the dataset into two parts. The previously mentioned parameters were recalculated for data below and above this threshold.

## 3. Results

Six days had to be excluded from further analysis because of data losses caused by technical issues and rainy weather conditions in September. As a result, data from 11 days of the study were available for analysis.

The inter-rater reliability between the two labelers was almost perfect, with κ = 0.99 for lying behavior and κ = 0.96 for rumination behavior. The correlation coefficients were interpreted according to Hinkle et al. [40].

### 3.1. One-Minute Time Resolution

For the analysis of data at a 1 min time resolution, five different datasets were available: two for rumination and three for lying behavior. The data were arranged and merged to ensure the largest possible sample size for each sensor system. Data losses occurred for various reasons and at different time points for each system, leading to slightly different sample sizes (Table 2).

According to IVO, cows were ruminating 12% of the time and lying 25% of the time across the datasets. The results of the confusion matrix and the correlation coefficients, calculated both with the original dataset and after applying the CA, are presented in Table 2.

The results for RWC1 and RWC2 showed no differences in either rumination or lying behavior. No results were available for RWC3 at a 1 min time resolution because this converter version does not offer behavior classification by the minute.

When comparing the results of the RWC versions with the ET for rumination times, the SE and SP of the RWC were both >90%, whereas the SE of the ET was considerably lower (83%) and that of the SP was higher (99%). The AC was similar in both systems, but the PPV was much lower for the RWC (60%) than for the ET (91%). Both correlation coefficients were high for the ET but only moderate for the RWC versions.

For the lying data at a 1 min time resolution, the LM1 demonstrated the best performance in classifying lying behavior according to the parameters presented. The results of the RWC versions of LM2 were slightly lower, but the parameters presented in Table 2 were still above 90% or 0.90, respectively. The ET had a very high SP and PPV, but the AC and r_S_ were both moderate, while the SE and CCC were low.

When applying the CA to the data from the ET and RWC, only minor changes were observed in the parameters for the ET (both rumination and lying behavior) and in the results for lying behavior of LM2 in both RWC versions (slight improvement). However, the CA had a considerable effect on the results for rumination of the NB in both RWC versions: the PPV increased from 60% to 87%, and both correlation coefficients rose from moderate to high correlation and agreement, respectively. The SE, SP, and AC for rumination also improved for the NB when using the CA.

### 3.2. Ten-Minute Time Resolution

The dataset at a 10 min time resolution included data from IVOs, the ET, RWC1 (NB and LM2), RWC2 (NB and LM2), RWC3 (NB and LM2), and LM1 (for lying behavior only). In total, 429 10 min intervals were available in the dataset for rumination, of which 509.6 min (12%) were classified as rumination according to the IVOs. The dataset for lying behavior contained 436 10 min intervals, with 1123.4 min (26%) classified as lying according to the IVOs. Neither rumination time nor lying time per 10 min interval was normally distributed. The R^2^, RMSE, and r_S_ for rumination and lying time per 10 min, with IVO as the gold standard, are presented in Table 3.

The correlation coefficients for rumination in the 10 min time resolution were very high across all sensor systems, with CCC > 0.9 and r_S_ ≥ 0.9. However, the most notable differences between the sensor systems were reflected in their R^2^ and RMSE results. Only the RWC3 had an R^2^ of >0.9 and an RMSE of <1 min, making it the best performer in classifying rumination time according to all parameters in Table 3, followed by RWC1/RWC2 and the ET. For lying time, no differences were observed among RWC1, RWC2, and RWC3. The three RWC versions demonstrated the best performance in classifying lying time, with very high correlation coefficients, closely followed by LM1. By contrast, the correlation coefficients for the ET were only moderate.

**Figure 1:**
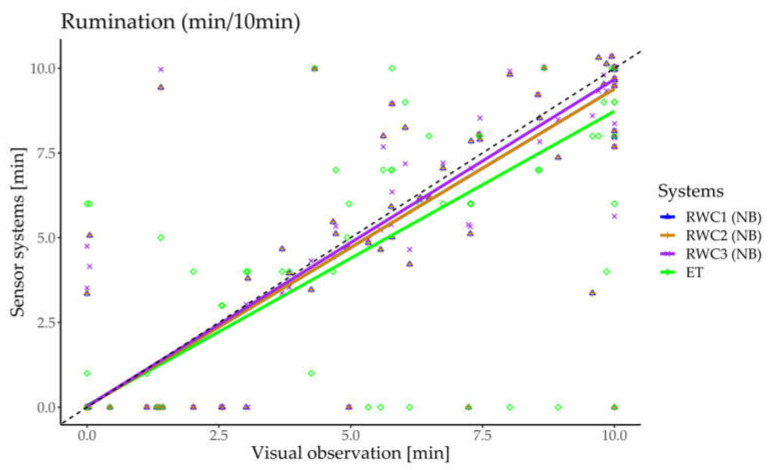
Comparison between the rumination time per 10 min classified by the different sensor systems (a noseband sensor (NB) with RumiWatch Converter versions V0.7.3.36 [RWC1 (∆)], V0.7.4.5 [RWC2 (+)], and V0.7.4.13 [RWC3 (×)]; and an ear-tag accelerometer (ET) system [ET (◊)]) and indirect visual observations (IVOs) based on 429 10 min intervals. The regression lines for RWC1 and RWC2 are identical. The dashed line represents the bisector.

Figure 1 shows the rumination time classified by the different sensor systems plotted against IVOs (i.e., the gold standard), along with the respective regression lines for each sensor system. The dashed line represents the bisector. Because the purple line is closest to the bisector, RWC3 can be interpreted as the system with the best performance, followed by both RWC1 and RWC2. Because the classification of rumination time by RWC1 and RWC2 is identical, their regression lines overlap. The ET (green rhombuses and line) showed lower agreement with the IVOs than the other systems.

The dataset included 235 10 min intervals with a THI below the threshold of 68 and 194 intervals with a THI of ≥68. All data collected in the evening hours were part of the 194 intervals with a THI of ≥68 (N = 97). According to the IVOs, rumination time was significantly higher (*p* < 0.001) when the THI was below the threshold. Table 4 presents the R^2^, RMSE, CCC, and r_S_ for rumination, listed separately for THIs of <68 and ≥68, enabling direct comparison of the sensors’ performance under different climatic conditions.

The correlation coefficients presented in Table 4 were higher when the THI was below the threshold of 68 for all systems. The RMSE values for the ET, RWC1, and RWC2 were lower when the THI was <68, but for RWC3, the RMSE was higher. According to the parameters presented, the performance of the ET was most affected by environmental conditions (high THI), while RWC3 was the least affected. There was no difference between RWC1 and RWC2, and both showed better results when the THI was <68. However, these results must be interpreted with caution, as half of the data at a THI ≥68 were collected in the evening hours, which could also influence cow behavior and eventually the sensors’ performance.

## 4. Discussion

### 4.1. Comparison of Different Sensor Systems

The results of this study reveal some differences in performance among the investigated systems across different time resolutions. Notably, there was no difference between RWC1 and RWC2 for rumination and lying behavior at both the 1 min and 10 min time resolutions. This indicates that the software changes between the two versions did not affect the classification of the behavioral categories “rumination” and “lying”, despite the implementation of some altered algorithms in RWC2 intended to optimize the detection of grazing behavior on pasture [22].

In our study, RWC3 performed best at a 10 min time resolution, followed by RWC1/RWC2 and the ET. In contrast, most of the performance parameters at the 1 min time resolution (Table 2) were highest for the ET, followed by the NB (RWC1/RWC2). However, applying the CA to 1 min data switched this ranking, which will be discussed in the next section. For classifying lying behavior, both LMs demonstrated very high agreement and correlations with the gold standard across all time resolutions, whereas the results for lying behavior from the ET were only moderate.

If sensors are attached to body parts that are directly involved in certain behaviors (such as legs for “lying” and “standing”), they might classify these behaviors more accurately than other systems, which are placed in a more neutral position (such as the neck or ear) and therefore need to infer indirectly to the behavior in question. Our data regarding the classification of “lying”, using two different LMs and one ET, support this assumption. Furthermore, these findings align with the report by Chapa et al. [7], who noted that the position of sensors on animals and the type of behavior being classified influence sensor systems’ performance. It might be worth to note when comparing these systems that both LMs are tightly attached to the legs, while the ET can turn around to a certain extent, which might challenge the algorithms that classify activity- and posture-related behavior even more. Moreover, not only the position on the animal but also the type of sensors can influence their performance for measuring or predicting certain parameters. The NB used in our project consists of a pressure sensor and an accelerometer to classify feeding-related parameters. In contrast, the ET relies on only acceleration data to classify rumination. However, this system also offers the feature of real-time localization, which is accomplished by the principle of triangulation. Analyzing localization data together with acceleration data could be beneficial for classifying “lying”, especially in an indoor setting. This was beyond the scope of the current study; however, other researchers have already suggested combining accelerometers with different other technologies to improve the classification of behaviors for which the detection performance is otherwise low [7]. In general, when combining several features in one sensor system, factors such as the additional energy consumption and potentially higher weight of the sensor need to be considered.

### 4.2. Correction Algorithm

Before applying the CA, the results for the NB (both RWC1 and RWC2) were surprisingly low at the 1 min resolution (CCC = 0.68) relative to the very high agreement with IVOs at the 10 min resolution (CCC = 0.94). Moreover, Werner et al. [18] reported an agreement of 91.1% for data at a 1 min time resolution using RWC V.0.7.3.36 (RWC1) for feeding behavior on pasture (which included grazing, ruminating, and other activities). When raw data are converted and consolidated into time resolutions of ≥10 min intervals, the RWC software performs validity checks to classify behavior as rumination only if there are >30 jaw movements per minute, sustained for ≥3 min with a steady frequency [17]. On this basis, we decided to apply the CA, which we typically used for 1 min time resolution data from LM1 [13,14], to the NB data as well. Ledgerwood et al. [14] demonstrated that using this type of CA yields more accurate results for classifying lying behavior with LM1. Because rumination also occurs in continuous bouts, and considering the validity checks performed by the RWC software, we hypothesized that applying the same CA used for LM1 data could improve the NB results at the 1 min time resolution.

While we acknowledge that rumination can occur for only ≤1 min—such as when a cow is interrupted for any reason—we consider these instances less likely than the misclassification of eating as rumination by the system. The findings of this study support this assumption because the results for RWC1 and RWC2 improved significantly after applying the CA to the 1 min time resolution data. Unexpectedly, the CA did not improve the results of the ET at the 1 min time resolution. This may suggest that the ET was missing entire bouts of rumination rather than misclassifying single minutes of rumination, as the CA would have corrected for the latter.

Applying the CA did not result in substantial changes for the classification of lying behavior, neither for the LM2 nor for the ET. Regarding the two different LMs, the CA has already been proven as beneficial in prior studies using LM1 for detecting “standing” and “lying” but did not improve the results of the LM2. One possible explanation for this could be found in the different logging intervals, as the LM1 was set to log once a minute and the LM2 had a logging frequency of 10 Hz. Leg movements, such as kicking within a minute, can be detected and taken into account with a high logging frequency, but lead to erroneous classification at a lower logging frequency when occurring at the moment the recording takes place. One possible explanation why the CA did not improve the results of the ET could be that this system was missing entire lying bouts rather than misclassifying single minutes, as described for the classification of rumination.

Further exploration of the usefulness of CAs at different time intervals could be beneficial if the NB is to be used for classifying rumination behavior at a 1 min time resolution. In general, this kind of CA might be applicable to any behavioral categories that usually occur in longer bouts, so it could be useful for researchers working with other sensor systems and animal species. We cannot draw conclusions on whether this CA might improve the classification performance of other ET systems at a 1 min time resolution, as their proprietary algorithms most probably differ from the ones used in our study.

### 4.3. Comparisons with Other Validation Studies

Compared with previous validation studies conducted under confined conditions [9,10], the ET showed lower agreement with visual observations in the current study. However, it is important to consider the time resolution of the data: the previous validations used a 1 h time resolution, whereas the current experiment utilized both 1 min and 10 min time resolutions. Additionally, we used the original algorithms developed for detecting rumination in cows in a barn environment without adapting them for use on pasture. This approach was chosen to facilitate comparison with other studies and to evaluate the system as it was designed. However, if more research were carried out with this ET system on pasture, it might be valuable to develop improved algorithms for that purpose. These findings can also be relevant for other ear-tag-based systems that were developed under housed conditions and could be used on pasture as well.

Compared with the results reported by Steinmetz et al. [22], who validated the NB using RWC V.0.7.4.5 (RWC2) at a 1 min time resolution under confined conditions and achieved an SE, SP, and AC of 95.3%, 92.3%, and 93.3%, respectively, our study yielded slightly lower performance (91.5%, 91.4%, and 91.4%, respectively). However, after applying the CA, the results improved to 93.6%, 98.1%, and 95.8%, respectively. To our knowledge, no other study has evaluated this converter version under grazing conditions for rumination or lying behavior.

Norbu et al. [20] conducted an experiment to assess the performance of RWC versions V.0.7.3.36 and V.0.7.4.13 under both confined and outdoor conditions using a 10 min time resolution. Although rumination behavior was not considered in their study because of insufficient observation periods, their findings on feeding-related parameters (prehension bites, mastication chews, and total jaw movements) indicated that RWC1 performed better than RWC3. Interestingly, in housed cows, RWC3 performed better than RWC1. On the basis of these results, it could be hypothesized that divergent classification of jaw movements may lead to varying performance between different converter versions and housing conditions in measuring rumination times. In our study, RWC3 performed better than RWC1 at a 10 min time resolution, whereas at the 1 min time resolution, we could not find a difference between RWC1 and RWC2. RWC3 does not offer the option to classify data at a 1 min time resolution.

In the current study, only minor differences were observed between RWC1 and RWC3 in most calculated parameters (CCC, r_S_, and RMSE), with the coefficient of determination for RWC1 showing high agreement (R^2^ = 0.88) and that for RWC3 showing very high agreement (R^2^ = 0.92) with the IVOs at a 10 min time resolution. The difference between these two converter versions was even more pronounced under heat stress conditions (R^2^ = 0.73 vs. R^2^ = 0.90). Li et al. [23] found very high correlation and agreement (r = 0.97, CCC = 0.96) between visual observations and the NB using RWC3 for identifying rumination chews per 10 min interval; however, they did not calculate rumination times to compare the performance at different time resolutions.

This study closes the gap in the direct comparison of the performance of the three latest converter versions in measuring rumination time in dairy cows on pasture. On the basis of the results, the appropriate converter version can be selected according to the required time resolution. For 10 min time resolutions, RWC3 is the best choice for measuring rumination. However, for a 1 min resolution, RWC1 or RWC2 may be preferable, especially when applying a simple CA, as used in this study. The choice of RWC version should also consider which other parameters (e.g., grazing) are of interest for a specific project. Regarding lying behavior, the three converter versions can be used interchangeably, according to our results.

### 4.4. Effects of Environmental Conditions on Sensors’ Performance

By dividing the 10 min dataset according to the environmental parameters of ambient temperature and relative humidity (converted into the THI), we tried to investigate the influence of weather conditions on the performance of the systems. Pinto et al. [39] reported that higher respiration rates in cows with a THI of ≥68, both in standing and lying positions. Although we did not measure the respiration rate in the current study, it seems reasonable that more frequent or pronounced respiration, or even panting, could affect the head and ear movements of cows, thereby influencing the classification accuracy of sensor systems. Additionally, Dougherty et al. [41] noted that head and ear movements in grazing cows increased with higher fly numbers. While we did not objectively measure fly load in this study, we observed more defensive movements involving the ears, head, and tail on hot summer days, leading us to hypothesize that these increased movements could account for the inferior performance of both systems during time intervals when the THI was ≥68.

The fact that the ET system’s performance was more strongly influenced by these environmental conditions than the NB slightly supports the assumption above. The classification of rumination based on patterns of regular ear movements could be more affected by defensive ear and head movements than classification based on pressure differences caused by chewing, measured directly at the nose ridge (as carried out by the NB). Notably, however, if a cow stops ruminating to chase away flies with its nose or to lick its body because of itching, the NB’s classification of rumination could also be impaired.

Weinert-Nelson et al. [21] investigated the performance of the NB using two different converter versions, V.0.7.3.2 and V.0.7.3.36 (RWC1), under thermoneutral and heat stress conditions in housed cows. Although they observed some differences in classification performance based on the type of behavior (eating, ruminating), converter version, and THI, they concluded that the NB could accurately quantify eating and rumination times in confined cattle, even under heat stress conditions. By contrast, our current data suggest an influence of heat stress on sensors’ performance, varying by sensor type (ear-tag vs. noseband) and converter version. The NB using RWC3 seemed most robust against heat stress conditions, with only a 3 percentage point reduction in CCC, whereas the ET was most impaired, showing a 24 percentage point reduction in CCC. Contrary to Weinert-Nelson et al. [21], our study observed a 14 percentage point reduction in CCC for RWC1. This difference may be attributed to the different housing conditions (pasture vs. confined), as heat stress may more significantly influence cows’ behavior on pasture due to factors like direct sunlight or flies, as previously mentioned. Additionally, minor differences in THI calculations and definitions of heat stress between studies could contribute to these variations. Furthermore, in our study, half of the data that were classified as THI ≥68 were recorded in the evening hours, after the evening milking. This was not carried out with the aim of comparing morning and evening hours but to collect more data in total. We have to acknowledge that a possible effect of the time of the day on cow behavior could also affect the sensors’ performance, but we consider our dataset to be not suitable for investigating the effect of the time of the day. Therefore, our findings regarding the sensors’ performance under heat stress conditions need to be interpreted with caution, as they might be influenced by the inclusion of evening hours.

In conclusion, it appears that the performance differences among the systems can be attributed to behavioral changes in the animals under heat stress, fly load, and probably also diurnal patterns of behavior. We did not investigate the performance of the sensor systems under housed conditions with and without heat stress and at different times of the day. Therefore, the findings of this study provide only initial insights into the performance of sensor systems under heat stress conditions on pasture. These results underscore the importance of validating sensors under different environmental conditions. This is a crucial consideration when conducting studies on heat stress in cows, especially when animal behavior is classified using PLF technologies.

### 4.5. Observation Strategy

The observation strategy using a drone to record video footage proved advantageous because it eliminated the need for installing stationary cameras on the pasture and minimized the influence of human presence on cow behavior. However, as previously mentioned, habituation to the drone was necessary to ensure that the cows’ behavior remained undisturbed, and this was conducted prior to the start of the experiment. Using a drone also allowed for quick and effective adjustments to the camera angle, which was particularly useful when the animals were moving or overlapping.

For analyzing the video footage, this observation strategy facilitated an efficient workflow because there was only one video to watch at a time, with all cows mostly visible in the same footage. The only exceptions occurred when it was not possible to adjust the camera angle quickly enough.

One shortcoming of this technique is that it did not allow continuous monitoring of the entire period the cows spent on pasture; this is because the drone’s batteries needed to be changed after approximately 20 min of flight under practical conditions (e.g., wind, the time for returning to landing place included some buffer). Continuously recording video footage would require at least two drones and two pilots, which was not feasible for this project.

In this project, indirect visual observation was used as the gold standard to classify animal behavior for evaluating different sensor systems attached to the animals. Another promising and non-invasive approach to classify animals’ behavior is the use of computer vision techniques, which have been developed and improved recently. However, at the start of this project, computer vision technology was not as widespread and advanced as today and it was not included as an objective of this study. Nevertheless, the data from different sources collected in this study might be useful for further projects dealing with computer vision approaches. Current challenges regarding the application of computer vision models in a dairy farm setting are, for example, individual animal identification, heterogeneous light conditions, and varying camera perspectives, often requiring finetuning of models if the viewing angle changes considerably. In the current study, the possibility of flexibly moving around with the drone while recording facilitated changing the camera’s perspective as needed for the subsequent labeling process by human observers, according to the movements of the cows on pasture. Recording the entire group of study cows together with one camera was the purpose of our observation strategy for the previously mentioned reasons. Consequently, a major part of the video footage of this study contains unfavorable conditions for training computer vision models in the first place, as the light conditions and camera perspectives change frequently.

One important topic that is currently addressed by many researchers in the field of computer vision is individual animal identification. In this project, identifying animals and subsequently assigning behaviors to each cow individually was crucial for the evaluation of the different sensor technologies. For the reliable identification of cows by the human observers, numbers were painted on their gluteal regions with hair dye; therefore, whether and to what extent this material is useful for projects on computer vision might mainly depend on the specific research question.

To summarize, using video recordings to label animal behavior manually served as the gold standard for the evaluation of three different on-cow sensors. Although having collected video footage and a corresponding labeled dataset in this project, we have not yet used it for research questions regarding computer vision. Nevertheless, especially because the conditions of our recordings may be challenging for a computer vision model, they might be helpful for other projects to test and further improve existing models.

### 4.6. Shortcomings

As previously mentioned, only 12% of the total analyzed data represented rumination time and 25% represented lying time. All systems showed very high specificity for both parameters, indicating that they performed well in detecting the absence of the behavior in question. However, when interpreting the correlation coefficients, it should be noted that the majority of the data consisted of “not ruminating” and “not lying”, which could bias these results.

Data loss was an issue that occurred to varying extents and for different reasons across the sensor systems. One significant weakness of this ET is its susceptibility to data loss due to the data transmission protocol and a lack of internal memory capacity. Raw data are sent in small packages wirelessly to the receivers using the 2.4 GHz frequency band. If any interference occurs, such as with the drone used in this study, the data package cannot be delivered or cached inside the ear-tag. Although the drone in this project also communicated using the 5.8 GHz frequency band, minimizing interference, this could still pose a data integrity issue on farms, where many devices operate on the 2.4 GHz band. Kulkarni et al. [42] discussed strategies for avoiding interference issues in wireless sensor networks, which could be relevant in this context.

Additionally, data loss from the ET occasionally occurred during the trial for several minutes whenever the connection between the ear-tags and receivers was impaired.

Regarding the NB and LM2, data loss is generally not a problem due to data transmission, as the raw data are stored on an internal micro-SD card. However, data loss can occur when the battery life is shorter than expected. Although the manufacturer states that the two combined 3.6-V lithium-metal (Li-SOCl_2_) batteries should last for approximately 100 days of raw data logging, in this study, battery life was approximately 40 days. This unexpected battery failure led to data loss on one of the experimental days for most of the sensors of the NB and LM2 system, resulting in the exclusion of that entire day’s data from analysis to keep the dataset balanced across all systems.

Several tasks can be carried out with the RumiWatch Manager software during raw data collection, such as checking the time synchronicity, verifying that the device is still logging, fetching summaries, and monitoring the live view of pressure differences measured by the pressure sensor and the tilt of the animal’s head measured by the accelerometer (the “animal live” view). These tasks vary in energy consumption, which can cause differing battery runtimes, depending on the intensity of monitoring during a measurement series. In this study, all RWS units were checked twice daily during the measurement periods, which might partly explain the shorter battery runtime. In general, the state-of-charge value of Li-SOCl_2_ batteries is hard to estimate, as, due to their flat discharge curve, Li-SOCl_2_ batteries will keep a constant voltage well after 90% of discharge [43]. Therefore, it is recommended to keep records of the number of days each battery has been used for logging to better estimate the remaining battery life. This is an important consideration when working with this system. Further technical studies on the energy consumption of the system under various conditions and management routines might help provide more accurate recommendations on battery lifetimes.

No data loss occurred for the LM1; data were successfully retrieved for all study periods. Nevertheless, data from specific days with extensive data loss from the other systems were also excluded from the LM1 datasets to avoid biased results due to major differences in sample size. Although the data loss in this study is a shortcoming, it also provides valuable experience in combining and handling sensor systems.

Because the study design focused on using different sensor technologies on pasture, there were no corresponding experimental periods under housing conditions. Therefore, we cannot provide a direct comparison between the performance of the different systems in the barn and on pasture. Additionally, the possibilities for interpreting the influence of heat stress on sensor-based classification of animal behavior are limited.

Understanding the capabilities and limitations of sensor systems is crucial for their application in research and practice. The new insights gained from this project on using sensor systems in dairy cows on pasture contribute valuable knowledge to the scientific field of evaluating PLF technologies.

## 5. Conclusions

This study evaluated three sensor systems for classifying rumination and lying behavior in dairy cows on pasture. We found that the position of the sensors on the animal, the behavior being monitored, and the time resolution of the classified data are crucial factors affecting the performance of a system. The results for lying behavior suggest that both LMs can be reliably used on pasture and should be preferred over the ET for measuring lying times in grazing dairy cows. For detecting rumination behavior, both the ET and the NB are generally suitable for use on pasture. However, our findings suggest that there might be an influence of heat stress on the sensors’ performance, which can differ among systems. Further studies on pasture should take heat stress and diurnal patterns of behavior into account.

According to our findings related to the 1 min time resolution data, we recommend applying a simple CA as described in this manuscript when working with rumination data from the NB, which may be relevant for future research projects. This study underscores the importance of validating sensors under different conditions and provides valuable insights into the factors influencing the performance of PLF tools. Further development of sensor systems should consider the broadest possible range of environmental conditions and aim for simplicity and reliability in their use. Additionally, further studies applying these technologies to investigate research questions on animal health, welfare, and sustainability in dairy farming are essential. This trend is already emerging in the field of PLF.

## Figures and Tables

**Table 1:** Ethogram for visual classification of cow behavior, derived from Schmeling et al. [26].

Classification	Description
**Feeding-related behavior**	
Ruminating	The animal regurgitates a food bolus and then chews and swallows it. The duration is calculated from regurgitating the first bolus to swallowing the last bolus.
Not ruminating	Every behavior other than ruminating.
Not analyzable	The animal is not visible in the video image, the animal is covered by another animal, or a lack of detail in the image does not allow for reliable classification of ruminating/not ruminating.
**Activity-related behavior**	
Lying	The body of the animal is not supported by any limb. The sternum and/or the belly are/is in contact with the ground. The limbs are bent or stretched out.
Not lying	The body of the animal is supported by at least two limbs. “Not lying” starts when the cow puts weight on the carpal joints and therefore initiates the process of standing up.
Not analyzable	The cow is not visible in the video footage.

**Table 2:** Sensitivity (SE), specificity (SP), balanced accuracy (AC), positive predictive value (PPV), Lin’s concordance correlation coefficient (CCC), and Spearman’s rank correlation coefficient (r_S_) for each of the systems (the ear-tag accelerometer (ET) system; a noseband sensor (NB) with a leg-mounted accelerometer (LM2) with RumiWatch Converter versions V0.7.3.36 (RWC1) and V0.7.4.5 (RWC2); and a HOBO-logger (LM1)) in comparison to indirect visual observations (IVOs) for the classification of rumination and lying behavior at a 1 min time resolution. The number of data points in each dataset (N) is provided in the last column. The results after applying the correction algorithm (CA) to the datasets are shown in italics beneath the original results for each system. The values most affected by the CA are highlighted in bold font. No additional results are provided for the LM1 because the CA is already integrated into the standard method for analyzing LM1 data.

Parameter and System	SE (%)	SP (%)	AC (%)	PPV (%)	CCC	r_S_	N (min)
**Rumination**							
ET	83.0	98.9	91.0	91.2	0.85	0.85	9682
*With CA*	*83.7*	*98.9*	*91.3*	*91.5*	*0.86*	*0.86*	
RWC1 (NB)	91.5	91.4	91.4	**60.1**	**0.68**	**0.70**	9667
*With CA*	*93.6*	*98.1*	*95.8*	***87.4***	***0.89***	***0.89***	
RWC2 (NB)	91.5	91.4	91.4	**60.1**	**0.68**	**0.70**	9667
*With CA*	*93.6*	*98.1*	*95.8*	***87.4***	***0.89***	***0.89***	
**Lying**							
ET	39.2	99.3	69.2	94.7	0.48	0.55	10,001
*With CA*	*38.7*	*99.3*	*69.0*	*94.7*	*0.47*	*0.54*	
RWC1 (LM2)	93.2	97.9	95.6	93.8	0.91	0.91	9740
*With CA*	*93.3*	*98.0*	*95.7*	*94.2*	*0.92*	*0.92*	
RWC2 (LM2)	93.2	97.9	95.6	93.8	0.91	0.91	9740
*With CA*	*93.3*	*98.0*	*95.7*	*94.2*	*0.92*	*0.92*	
LM1	0.96	99.1	97.6	97.3	0.96	0.96	10,097

**Table 3:** Coefficient of determination (R^2^), root mean square error (RMSE), Lin’s concordance correlation coefficient (CCC), and Spearman’s rank correlation coefficient (r_S_) of each system (an ear-tag accelerometer (ET) system; a noseband sensor (NB) with a leg-mounted accelerometer (LM2) with RumiWatch Converter versions V0.7.3.36 (RWC1), V0.7.4.5 (RWC2), and V0.7.4.13 (RWC3); and a HOBO-logger (LM1)) for rumination and lying time per 10 min compared with indirect visual observations (IVOs).

Parameter and System	R^2^	RMSE	CCC	r_S_
**Rumination**				
ET	0.83	1.24	0.91	0.90
RWC1 (NB)	0.88	1.01	0.94	0.90
RWC2 (NB)	0.88	1.01	0.94	0.90
RWC3 (NB)	0.92	0.82	0.96	0.92
**Lying**				
ET	0.44	3.33	0.55	0.66
RWC1 (LM2)	>0.99	0.16	>0.99	0.99
RWC2 (LM2)	>0.99	0.16	>0.99	0.99
RWC3 (LM2)	>0.99	0.16	>0.99	0.99
LM1	0.99	0.42	0.99	0.97

**Table 4:** Coefficient of determination (R^2^), root mean square error (RMSE), Lin’s concordance correlation coefficient (CCC), and Spearman’s rank correlation coefficient (r_S_) of each system (an ear-tag accelerometer (ET) system) and a noseband sensor (NB) with RumiWatch Converter versions V0.7.3.36 (RWC1), V0.7.4.5 (RWC2), and V0.7.4.13 (RWC3)) for rumination. The parameters are shown separately for a temperature–humidity index (THI) of <68 and ≥68 for a direct comparison.

Parameter and System	THI	R^2^	RMSE	CCC	r_S_
ET	<68	0.90	1.06	0.95	0.95
	≥68	0.59	1.42	0.71	0.71
RWC1 (NB)	<68	0.94	0.89	0.97	0.95
	≥68	0.73	1.14	0.83	0.75
RWC2 (NB)	<68	0.94	0.89	0.97	0.95
	≥68	0.73	1.14	0.83	0.75
RWC3 (NB)	<68	0.93	0.90	0.97	0.94
	≥68	0.90	0.70	0.94	0.83

## Data Availability

The raw data supporting the conclusions of this article will be made available by the authors on direct request.

## Supplementary Materials

The following supporting information can be downloaded at https://www.mdpi.com/article/10.3390/s24237739/s1. Figure S1: SMARTBOW system. Figure S2: HOBO-loggers. Figure S3: RumiWatch system.
