# Supplementary figures and images for: Evaluation of Different Sensor Systems for Classifying the Behavior of Dairy Cows on Pasture
Source: Sensors (Basel). 2024 Dec 3;24(23):7739. doi: 10.3390/s24237739 (PMC11644843; doi:10.3390/s24237739)

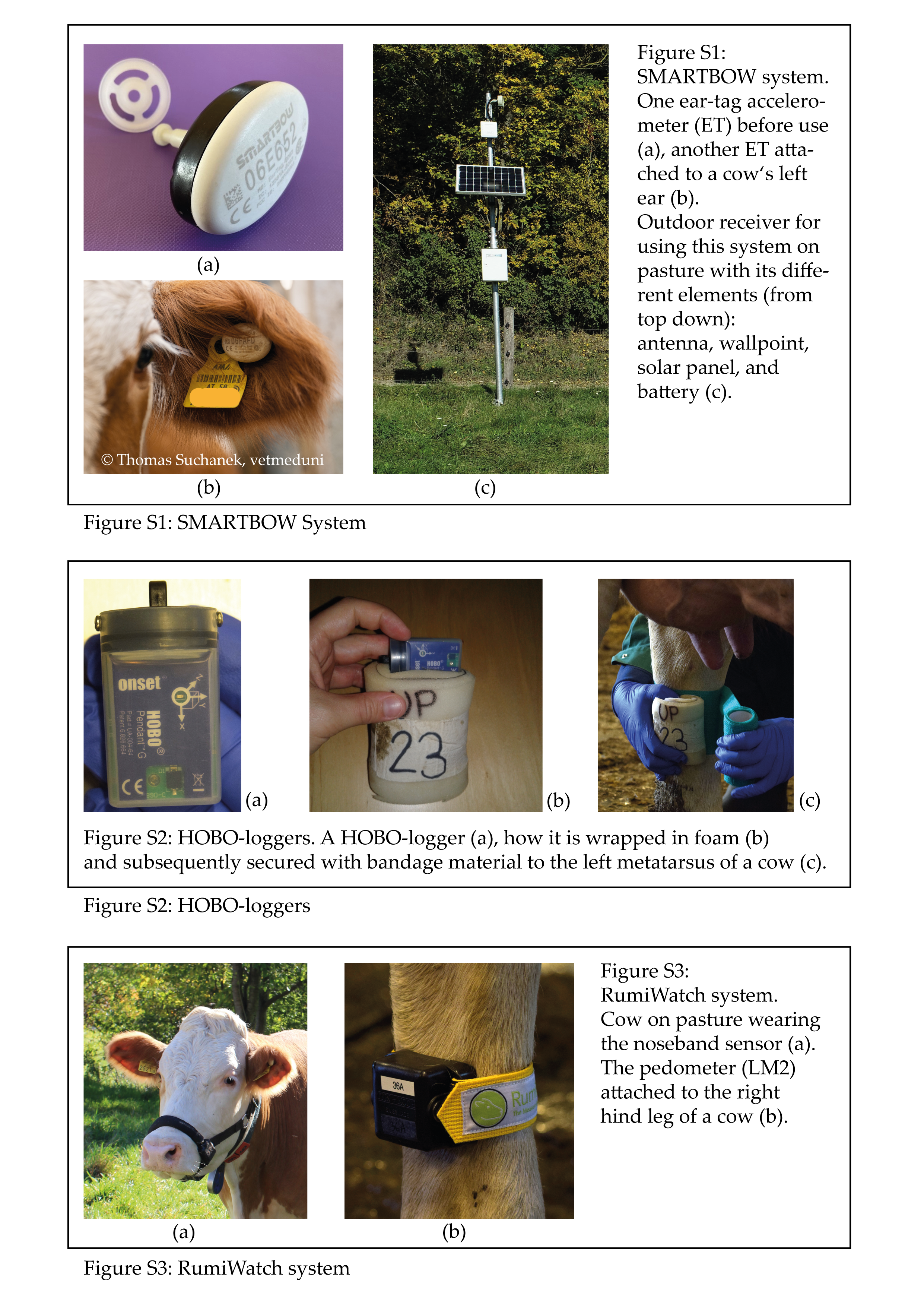

Supplement: Supplementary file 1 [file sensors-24-07739-s001.zip › sensors-3232804-supplementary.png]
